# Cranberry Polyphenols: Natural Weapons against Dental Caries

**DOI:** 10.3390/dj7010020

**Published:** 2019-03-01

**Authors:** Nebu Philip, Laurence J. Walsh

**Affiliations:** The University of Queensland School of Dentistry, Herston, Brisbane QLD 4006, Australia; l.walsh@uq.edu.au

**Keywords:** cranberry, Polyphenols, dental caries

## Abstract

Bioactive polyphenol components of cranberry (*Vaccinium macrocarpon*) are known to have virulence attenuating effects against several cariogenic virulence properties responsible for dental caries pathogenesis. In particular, cranberry A-type proanthocyanidins and flavonols have demonstrated potent inhibitory effects against cariogenic virulence targets such as bacterial acidogenicity, aciduricity, glucan synthesis, and hydrophobicity. Cranberry phenols have the ability to disrupt these cariogenic virulence properties without being bactericidal, a key quality essential for retaining the benefits of the symbiotic resident oral microbiome and preventing the emergence of resistant microbes. This review discusses the cariostatic mechanisms of specific cranberry phytochemicals and their potential use as therapeutic agents against cariogenic bacteria in the prevention and control of dental caries.

## 1. Introduction

There has been an increasing global interest in the potential therapeutic uses of plant-derived natural products to prevent oral diseases. Phytodentistry is based on long-standing evidence showing population groups that habitually consume polyphenol-rich diets have significantly better oral health [1]. Traditional chemotherapeutic approaches to oral diseases using synthetic oral biocides like chlorhexidine are becoming less popular because of the growing concerns of allergic reactions and the emergence of microbial resistance [2,3]. Furthermore, the realisation of the key health benefits of having an oral microbiome that is in a symbiotic relationship with the host has resulted in virulence-targeted therapies being preferred over broad-spectrum antimicrobials. This is especially vital for biofilm-mediated diseases like dental caries, where reversing the microbiome dysbiosis responsible for caries development is crucial for long-term disease control rather than simply eliminating the dental plaque biofilm [4].

The Global Burden of Diseases report highlighted that dental caries is the most common human disease condition worldwide [5]. Newer and more effective preventive measures that go beyond using fluoride dentifrices and conventional mechanical plaque removal are clearly needed to tackle this ubiquitous human disease. In this regard, a number of cariostatic natural products have shown the potential to be useful for dental caries prevention [6]. Phytochemicals present in the cranberry fruit are particularly promising, having been shown to exert biological effects against critical virulence properties of cariogenic bacteria, besides having other well-established benefits to human health. 

## 2. Origin and Health Benefits of Cranberry

Cranberry is a small red fruit that is cultivated almost exclusively in the cooler climes of the American Northeast, Canada, and Chile. Four species have been described: *Vaccinium macrocarpon* (large cranberry, American cranberry), *Vaccinium microcarpom* (small cranberry), *Vaccinium oxycoccus* (common cranberry or northern cranberry), and *Vaccinium erythrocarpum* (southern mountain cranberry) [7]. The American cranberry has received the most attention for its beneficial effects on human health dating back to the 17th century. Native Americans used cranberries as a meat preservative and a medicine to relieve diverse ailments from scurvy to stomach and liver problems. Today, cranberry is commonly recognised as having a preventive effect on urinary tract infections and gastric ulcers because its high-molecular-weight phytochemicals inhibit the adhesion of uropathogenic P-fimbriated *Escherichia coli* to the mucosa of the urinary tract and *Helicobacter pylori* to the gastric mucosa [8,9]. These anti-adhesive properties of cranberry phenols are unique and are not found with the polyphenols present in other fruits like grapes, apples, and oranges [10]. Other purported health benefits of cranberry include lowering cardiovascular and neurological disease risk, modulation of inflammatory responses, and inhibiting cancer cell proliferation [11,12].

With regards to oral health, in vitro studies have shown that cranberry constituents can exert beneficial effects on gingival and periodontal health by inhibiting the host inflammatory response, suppressing bacterial biofilm formation, and reducing the activity of periopathogenic proteolytic enzymes [7,13,14]. Specific flavonoids present in cranberry were also shown to disrupt key virulence factors responsible for dental caries pathogenesis [15,16,17]. This narrative review will explore the potential therapeutic targets and the specific cranberry phenols reported to be involved in disrupting the virulence traits of cariogenic bacteria.

## 3. Potential Cariogenic Virulence Targets

Dental caries is a polymicrobial multifactorial disease that develops when the acidogenic and aciduric members of the resident oral flora obtain a selective ecological advantage over other commensal microorganisms, disrupting the homeostatic balance of the plaque biofilm, and initiating the disease process [18]. While the disease is polymicrobial in nature, the mutans streptococci (MS) function as keystone pathogens, as they are principally responsible for the initial assembly of the exopolysaccharide (EPS) glucan-rich scaffold matrix. The ability of MS (predominantly *Streptococcus mutans* and *Streptococcus sobrinus*) to synthesise copious amounts of insoluble glucans is critical for caries pathogenesis [19]. The first part of this review discusses the cariogenic virulence traits that cranberry flavonoids can potentially target in order to reverse the dental plaque dysbiosis responsible for caries development.

### 3.1. Glucan Synthesis

The *S. mutans* glucosyltransferases (Gtfs) are exoenzymes that are primarily responsible for orchestrating the virulent plaque biofilm matrix. In the presence of sucrose, different Gtfs (i.e., B, C, and D) synthesise extracellular carbohydrate polymers called glucans. These glucans confer structural integrity and bulk to plaque biofilms, enhance bacterial adhesion and accumulation, and provide a readily metabolisable carbohydrate source for sustained acid production [20]. The glucan-rich EPS matrix also creates a diffusion limiting barrier that not only protects plaque microflora from topical antimicrobial agents but also helps maintain acidic microenvironments critical to enamel dissolution [21]. As the different Gtfs acting in concert are a primary virulence property of *S. mutans*, targeting Gtf enzyme activity should significantly disrupt the development of dental caries [22].

### 3.2. Acidogenicity

An important attribute of cariogenic microorganisms is their proficiency in producing large amounts of organic acids as a by-product of carbohydrate metabolism. These acids (particularly lactic acid) lower the pH of the plaque fluid, causing demineralization of the tooth structure and lesion initiation. Sustained low pH within the plaque biofilm will favour the survival and growth of acidogenic and aciduric bacteria, which start to dominate the plaque microbial community, displacing many of the health-associated commensal oral microflora [4]. Disrupting bacterial metabolism and acid production is thus key to not only preventing tooth demineralisation but also to restoring the ecological balance of dental plaque.

### 3.3. Aciduricity

The ability to survive and thrive in low pH environments is a key characteristic of MS. Proton translocating F_1_F_0_-ATPases play a major role in protecting the bacterial glycolytic and sugar transport system enzymes from the environmental stress caused by biofilm acidification. Inhibiting the F_1_F_0_-ATPase proton pump can result in cytoplasmic acidification and thus affect the optimal functioning of the intracellular acid-sensitive glycolytic enzymes.

### 3.4. Bacterial Adhesion

The *S. mutans*-derived Gtfs (particularly GtfC) are adsorbed onto salivary pellicle-coated tooth surfaces and rapidly generate an amorphous glucan layer that facilitates initial bacterial adhesion. Gtf exoenzymes (particularly GtfB) also have great affinity for cell surfaces of other microorganisms, converting them into de facto glucan producers in the presence of sucrose. The glucans formed in situ provide enhanced binding sites for MS, resulting in further bacterial aggregation, and favouring cohesive microcolonies [23]. While the Gtf-derived glucans are mainly responsible for MS adhesion and accumulation, cell hydrophobicity can also play a role in the initial bacterial adhesion to the salivary pellicle. The hydrophobicity of *S. mutans* is believed to be mainly associated with its cell surface proteins [24]. Targeting Gtf exoenzymes and bacterial surface hydrophobicity could therefore impede microbial adhesion and lower the virulence of dental plaque.

## 4. Cariostatic Effects of Cranberry Polyphenols

The cranberry fruit is a unique and rich source of bioactive polyphenol compounds. Among the different classes of polyphenols, the flavonoid family has the largest variety of compounds with pharmacological activity. The three major flavonoids found in whole cranberries are the flavonols, anthocyanins, and the flavanols that include monomeric flavan-3-ols and the polymeric flavan-3-ols called proanthocyanidins [25]. Very minute quantities of other polyphenols may also be found in cranberries including flavanones (e.g., prunin), non-flavonoids such as phenolics acids (e.g., benzoic acid), and anti-oxidant stilbenes (e.g., resveratrol) [11]. Table 1 below summarises the bioactive phytochemicals in cranberry fruit and their reported cariostatic effects. The role that specific cranberry flavonoids play in disrupting cariogenic virulence is discussed further below.

### 4.1. Flavonols

Cranberry flavonols occur mainly as glycosylated forms of quercetin, myricetin, and kaempferol [26]. The total flavonols in cranberry has been quantified to be approximately 300–500 mg/kg of the fruit [27]. The content and nature of cranberry flavonols are unique in character and are not usually seen in flavonols of other fruits. Cranberry flavonols are believed to be responsible for many of the systemic health benefits associated with cranberry consumption [11].

Cranberry flavonols, especially the most abundant quercetin glycosides, were found to be significant inhibitors of GtfB, GtfC, F_1_F_0_-ATPase, and of cytoplasmic glycolytic enzymes within *S. mutans* [15,16]. Flavonol-mediated Gtf inhibition appears to be related to its unsaturated double bond between C2 and C3, as anthocyanins that lack the C2–C3 double bond exhibited only modest inhibitory activity against glucan synthesis [15]. Quercetin is also known to be a non-competitive inhibitor of the proton-translocating F_1_F_0_-ATPase activity that is critical to the survival of acidogenic bacteria in the low pH biofilm environment they create [28].

### 4.2. Proanthocyanidins (PACs)

PACs (also referred to as condensed tannins) are oligomers or polymers of flavan-3-ols that are distinctive in terms of their size, high molecular weight, and free hydroxyl groups. These structural properties determine their bioactivity, including the ability to precipitate or denature polypeptides and proline-rich proteins [29]. Binding of PACs to salivary proteins is responsible for the astringent taste of cranberries and other tannin-rich foods. PAC subunits (catechin, epicatechin, epigalloatechin, epigalloatechin gallate) are linked most often via a single B-type bond and less often by a double interflavan A-type linkage. The A-linked PACs (A-PACs) are found in high concentrations in cranberry and can also be found in peanut skins and cinnamon [30,31]. The cranberry A-PACs are mainly composed of epicatechin monomer subunits with at least one A-type interflavan bond, and the type of PAC linkage is closely related to its biological effects. The A-PACs appear to be more biologically active than B-type PACs due to the conformational rigidity afforded by the double interflavan linkages [8,30]. For instance, A-PACs demonstrated significant anti-adhesion effects against *E. coli* and *H. pylori*, whereas B-type PACs were devoid of anti-adhesion properties. 

Among the different cranberry flavonoids, the A-PACs have exhibited the most potent effects against bacterial glycolytic, Gtf, and F_1_F_0_-ATPase enzymes [15,32]. The ability of PACs to form protein-polyphenol complexes possibly affects the enzyme activity by irreversibly binding to the catalytic and glucan-binding domains once complexed [32]. The precise mechanisms by which cranberry A-PACs inhibit *S. mutans* enzyme activity are likely to be related to their unique rigidity-conferring A-type linkages, as PACs from non-cranberry fruits that lacked A-type linkages did not affect these enzymes [16,17,32].

The PACs in cranberry are predominantly found in oligomeric forms (up to 13 monomeric units), although smaller PAC dimers are also seen. The bioactivity of the A-PACs are also strongly associated with their degree-of-polymerisation (DP). A-PACs with DP 4 and DP 8 to 13 are optimal for interaction and inhibition of Gtfs and glucan-mediated adhesion [17]. On the other hand, inhibitory activity against bacterial acid production is mostly related to a low molecular weight A-PAC dimer called procyanidin A_2_ [16]. It is unlikely that the larger PAC oligomers can directly affect intracellular bacterial glycolytic enzymes, though they may still have deleterious effects on membrane components of the glycolytic pathway [1].

Cranberry A-PACs have been shown to significantly reduce the incidence and severity of smooth surface caries in an animal caries model, suggesting they may have value as a supplement to fluoride for dental caries prevention [32]. Overall, the evidence does indicate that A-PACs have the most effective cariostatic properties amongst the different cranberry flavonoids, with the potential to be developed as natural agents for dental caries prevention.

### 4.3. Anthocyanins (ACYs)

ACYs such as cyanidin and peonidin are present at concentrations of approximately 100–900 mg/kg of the fruit and are responsible for the distinctive red colour of the fruit [33]. While ACYs may a have a number of systemic health benefits, their ability to inhibit bacterial cariogenic virulence properties are relatively low. A study comparing the effects of different cranberry fractions on virulence traits of *S. mutans* found that A-PACs exhibited the greatest biological activity, followed by the flavonols, while the ACY extract did not have any significant effects [15].

### 4.4. Nondialysable Material (NDM) 

A number of studies have reported beneficial effects from a high molecular weight fraction of cranberry that remains after dialysis of concentrated cranberry juice using membranes with a molecular weight cut-off of 12,000–15,000 [11]. Chemical analysis of the NDM fraction suggests it is a heterogeneous mixture of phenols containing about 65% PACs and trace amounts of ACYs [34].

A mouthwash supplemented with NDM has been found to significantly reduce salivary MS counts following six weeks of usage [35]. In vitro experiments have revealed that NDM inhibits the adhesion of *S. mutans* and *S. sobrinus* and promotes desorption of the bacteria from biofilms [35,36,37]. This is likely because NDM components may interact with bacterial cell surface hydrophobic proteins, reducing hydrophobicity and thus initial bacterial adhesion to tooth surfaces [37]. Cranberry juice itself may not be suitable for dental caries prevention as it has a high content of enamel eroding organic acids (e.g., quinic, citric, and malic acids) compared to juices from apple, grape, or blueberry. However, the potential of using the non-fermentable NDM fraction from cranberry juice concentrate in oral care products needs to be explored [13].

## 5. Cranberry vs. Other Natural Products

Cranberry polyphenols may have a number of advantages over other suggested cariostatic natural products. A comparison of three dark-coloured berries (cranberry, wild blueberry, and strawberry) showed that cranberry extracts had the greatest effects against *S. mutans* biofilm virulence properties. While tea (*Camellia sinensis*) also contains high levels of biologically active phenolic compounds, studies have shown cranberry polyphenols have the ability to effect a more powerful disruption of cariogenic virulence factors [1]. The greater cariostatic effects of cranberry could partly be attributed to the presence of the unique cranberry A-PACs that are not present in other polyphenol-rich plants.

Isolated phytochemicals like xanthorrrhizol from Javanese turmeric (*Curcuma xanthorrhizha*) and macelignan from nutmeg (*Myristica fragrans*) have also shown significant growth inhibitory effects against oral bacteria [38,39,40]. However, they lack the ability to discriminate between health- and disease-associated plaque microorganisms, which is key for long-term control over biofilm-mediated oral diseases like dental caries [40]. Cranberry polyphenols, while lacking bactericidal effects, have the potential to modulate cariogenic virulence, allowing them to possibly reverse the microbiome dysbiosis responsible for dental caries and still retain the key benefits of the resident oral microbiome.

A common problem with using natural products as therapeutic agents is their compositional variability. In this regard, cranberry is a particularly feasible and sustainable source of standardised bioactive compounds, as it is a chemically and genetically well-characterised fruit, with highly standardised methods for extracting the biologically active components [32].

## 6. Conclusions

Cranberry polyphenols have enormous potential for development as adjunctive anti-caries agents. However, the cariostatic effects of cranberry have mostly been demonstrated in laboratory studies. There is a need for well-designed clinical trials to evaluate whether the proposed cariostatic effects of cranberry phenols can actually translate into preventing dental caries in high-risk individuals and population groups. The combination of virulence-attenuating standardised cranberry extracts and remineralising agents in a single oral care product could hold great promise in the global fight against dental caries.

## Figures and Tables

**Table 1:** Cranberry polyphenols and their cariostatic effects.

Polyphenol Class	Cariostatic Effects
**Flavonoids**	
1. Flavonols e.g., quercetin, myricetin, kaempferol	Inhibits Gtf, F-ATPase enzyme activity and acid production
2. Flavanols	
(a) Monomeric flavan-3-ols e.g., catechin, epicatechin, epigallocatechin, epigallocatechin gallate	No reported effects
(b) Polymeric flavan-3-ols e.g., A-linked proanthocyanidins	Inhibits Gtf, F-ATPase enzyme activity and acid production
3. Anthocyanins e.g., cyanidin, peonidin, malvidin, delphinidin	Insignificant effects against cariogenic virulence factors
4. Flavanones e.g., prunin	No reported effects
**Nondialysable Material**	Interferes with bacterial hydrophobicity and initial stages of plaque development
**Phenolic acids** e.g., benzoic acid, ellagic acid	No reported effects
**Stilbenes** e.g., resveratrol	No reported effects
